# Diagnosis and treatment of iron deficiency in chronic heart failure: Position statement of the heart failure working group of the Austrian Society of Cardiology

**DOI:** 10.1007/s00508-025-02521-x

**Published:** 2025-05-06

**Authors:** Moritz Messner, Gerhard Pölzl, Christopher Adlbrecht, Johann Altenberger, Johann Auer, Robert Berent, Jakob Dörler, Marc-Michael Zaruba, Christian Ebner, Friedrich Fruhwald, Martin Hülsmann, Deddo Mörtl, Peter P. Rainer, Anna Rab, Thomas Weber, Rudolf Berger, The Heart Failure Working Group of The Austrian Society for Cardiology

**Affiliations:** 1 Department of Internal Medicine III, Medical University Innsbruck, Innsbruck, Tyrol Austria; 2 Imed19-privat, private clinical research center, Chimanistrasse 1, 1190 Vienna, Austria; 3 Center for Cardiovascular Rehabilitation, Lehrkrankenhaus der PMU, Pensionsversicherung Grossgmain, Grossgmain, Austria; 4 Department of Cardiology and Intensive Care, St Josef Hospital, Braunau, Upper Austria Austria; 5 Center for Cardiovascular Rehabilitation, HerzReha Bad Ischl, Bad Ischl, Upper Austria Austria; 6 Department of Internal Medicine and Cardiology, Landeskrankenhaus Klagenfurt, Klagenfurt, Carinthia Austria; 7 Second Medical Department, Convent Hospital Elisabethinen, Linz, Upper Austria Austria; 8 Department of Internal Medicine, Division of Cardiology, Medical University Graz, Graz, Styria Austria; 9 University Clinic of Internal Medicine II, Department of Cardiology, Medical University Vienna, Vienna, Austria; 10 Department of Internal Medicine 3, University Hospital St. Poelten, Karl Landsteiner Private University, St. Poelten, Lower Austria Austria; 11 Department of Internal Medicine, St. Johann in Tirol General Hospital, St. Johann in Tirol, Austria; 12 University Heart Center, Medical University of Graz, Graz, Austria; 13 BioTechMed Graz, Graz, Austria; 14 Department Internal Medicine I, Kardinal Schwarzenberg Klinikum, Schwarzach, Austria; 15 Department of Cardiology, Klinikum Wels-Grieskirchen, Wels-Grieskirchen, Upper Austria Austria; 16 First Medical Department, Hospital of St. John of God, Eisenstadt, Burgenland Austria

**Keywords:** Iron deficiency, Heart failure, Austrian consensus statement, Iron supplementation, Iron derisomaltose, Iron carboxymaltose

## Abstract

Iron deficiency (ID) is a common comorbidity in heart failure (HF), affecting 55% of chronic and up to 80% of acute HF patients, regardless of ejection fraction (EF). An ID is associated with reduced quality of life, impaired exercise capacity (VO_2_ peak), higher hospitalization rate and lower survival rate. It is also an independent predictor of HF outcomes. This consensus statement critically reviews the diagnostic criteria for ID in HF and provides recommendations for their use. The efficacy and safety of intravenous iron supplements, including ferric carboxymaltose (FCM) and ferric derisomaltose (FDI), are analyzed highlighting the indications and potential adverse effects. Key clinical trials and guideline recommendations are summarized. In summary, the document addresses the diagnostics, treatment and monitoring of ID in HF.

## Introduction

Iron deficiency (ID) is one of the most common conditions in patients with heart failure (HF), affecting approximately 55% of patients with chronic HF and up to 80% of patients with acute HF, irrespective of the ejection fraction (EF). In HF patients, ID is associated with reduced quality of life (QoL), exercise capacity (as peak VO_2_) and survival as well as an increased risk of hospitalization. The presence of ID is a strong independent predictor of HF outcome [1] and thus independent of demographic and clinical variables, including anemia [2].

This document discusses criteria and expert opinions on the diagnosis of ID in HF, the benefits of IV supplementation with ferric carboxymaltose (FCM) and ferric derisomaltose (FDI) and the monitoring of patients with ID in HF.

## Physiological role of iron and pathophysiology of ID

The human body contains about 3–5 g of iron, which the body requires for the synthesis of hemoglobin. In addition to the transport of oxygen in the blood, iron as a central trace element is essential for the functionality of numerous other intracellular and mitochondrial processes in the respiratory chain. Iron is also involved in the synthesis and degradation of proteins, lipids and nucleic acids [3–5]. Especially in highly oxygen-consuming cells, such as cardiomyocytes and proximal renal tubular cells, iron is crucial for proper functionality [6].

The daily absorption of iron in the duodenum typically ranges from 1–3 mg. Given that ferrous iron (Fe^2^⁺) is highly reactive and cytotoxic, leading to oxidative damage and to a specialized form of iron-dependent programmed cell death known as ferroptosis, complex regulatory mechanisms are essential for maintaining iron homeostasis [7]. On absorption in the duodenum, Fe^2^⁺ is stored primarily in reticuloendothelial cells and the liver. Hepcidin, a hormone produced by the liver, plays a crucial role in regulating systemic iron levels by modifying duodenal absorption of iron, inducing the degradation of ferroportin, the iron exporter on cellular membranes, thereby regulating iron release into the bloodstream [8].

In the circulation, ferric iron (Fe^3^⁺), which is less reactive, binds to transferrin. The iron-transferrin complex is internalized into cells through receptor-mediated endocytosis via the transferrin receptor protein (TfR1). Once inside the cytoplasm, Fe^3^⁺ is reduced to Fe^2^⁺ for cellular utilization [7].

## Absolute and functional ID

Patients with HF can present with absolute or functional ID. In absolute ID, iron stores are depleted by loss (bleeding) or reduced uptake (malabsorption or dietary shortage). Absolute ID occurs in patients with HF due to occult gastrointestinal bleeding with or without antiplatelet or anticoagulant treatment [9] or due to reduced uptake when gastric acidity is reduced by proton pump inhibitor treatment [10]. Calcium channel blockers can disrupt the function of membrane-bound channels essential for iron uptake by enterocytes [11]. Chronic inflammation as well as hepatic congestion due to increased central venous pressures upregulates the expression of hepcidin, which further inhibits duodenal absorption of dietary iron [12]. In elderly HF patients, inadequate dietary iron intake may occur due to anorexia, while iron losses may be exacerbated by underlying gastrointestinal malignancies [13–15].

In functional ID, iron cannot be effectively mobilized from existing stores to meet the needs of tissues, leading to functional impairments in processes, such as cardiomyocyte function, skeletal muscle activity and erythropoiesis. Although there is sufficient iron in the body, it cannot be adequately released from the reticuloendothelial system (RES). Hepcidin serves as an important regulator of plasmatic iron homeostasis. Iron deficiency and erythropoietic stimulation suppress hepcidin production to provide adequate iron quantities [16, 17].

In contrast, inflammation, such as chronic inflammation in HF, induces hepcidin production [8, 18]. Elevated levels of hepcidin elicit functional ID, characterized by a reduction in transferrin-bound iron (transferrin saturation, TSAT). This results in a limited iron supply to cells. Inflammation leads to increased secretion of intracellular ferritin into the bloodstream, highlighting its role as an acute phase reactant. Consequently, higher serum ferritin level cut-offs are used for diagnosing ID in HF patients, who experience chronic inflammation [19, 20].

A simplified overview of the constellation of important parameters in absolute and functional ID is provided in Table 1. For the sake of completeness, it should be mentioned that both conditions may be present at the same time.

**Table 1:** General overview of the laboratory parameters in ID based on [46]

Parameter	Absolute ID	Functional ID	Combined ID
Transferrin	↑	↓/⊥	↓
TSAT	↓	↓	↓
Serum iron	↓	↓	↓
sTfR	↑	⊥	⊥/↑
Ferritin	↓	⊥/↑	↓/⊥
Inflammatory markers	⊥	↑	↑

*TSAT* transferrin saturation, *sTfR* soluble transferrin receptor, ↓ reduced, ↑ increased, ⊥ normal

## Diagnosis of ID in HF

In HF the presence of ID (absolute or functional) affects cardiomyocytes and skeletal muscle, impairing functional capacity. The clinical manifestations of ID range from lethargy, weakness, fatigue, brittleness of the fingers, pallor of the skin and mucous membranes, to the presence of rhagades at the corners of the mouth [21].

Patients with HF often have functional ID [4]. Diagnostic criteria of functional ID in HF are commonly not based on clinical symptoms but on reduced TSAT (an indicator of low iron levels in the blood), with serum ferritin levels that rule out absolute ID [22]. The arbitrary higher serum ferritin threshold of 100 μg/L, in contrast to 30 μg/L in the general population, is used to identify absolute ID in HF due to increased inflammatory activity. It is important to note, that this cut-off point was never tested in HF patients.

The diagnostic approach to ID should identify patients who may benefit from iron supplementation in hard outcomes, such as all-cause mortality and heart failure hospitalization, as well as functional parameters and quality of life.

The normal range of serum ferritin is wide (≈ 20–300 μg/L), making it difficult to distinguish mild to moderate inflammation-related increases from normal levels in healthy adults. Thus, a serum ferritin level > 20–30 μg/L is not suitable to diagnose functional ID but is suitable to exclude absolute ID. When levels exceed 400–500 μg/L in functional ID, the serum ferritin level indicates iron overload [23].

As increased ferritin is also caused by inflammation, a central trigger of HF, low levels can also be a biomarker of stable conditions. In contrast, high levels of ferritin can mirror advanced HF stage and not iron overload. This double-edged sword makes interpretation difficult in all acute and chronic inflammatory processes.

In ID patients cardiomyocytic transferrin receptor 1 (TfR1) expression is high because cells are trying to take up more circulating iron when cytosolic levels are low. Independent of systemic iron, in failing hearts myocardial iron storage, mitochondrial activity, hepcidin and soluble transferrin receptor (sTfR) are decreased, whereas ferroportin is increased, indicating reduced entry and increased egress of iron from cardiomyocytes [6, 24]. Chronic sympathetic stimulation and high plasma norepinephrine are linked to cardiac iron depletion and lower sTfR and TSAT levels in HF patients [25]. Functional ID, characterized by low TSAT, increased hepcidin and normal range serum ferritin, indicates poor response to oral iron in HF. Serum ferritin and hepcidin are less reliable for identifying ID due to various influencing factors, while more suitable sTfR levels, although linked to poor outcomes, are not yet standardized or widely used in clinical trials [23].

Serum ferritin (iron storage) and TSAT (iron mobilization) are considered suitable for assessing the iron status in chronic diseases. The current guidelines from the European Society of Cardiology (ESC) recommend identification of ID in patients with HF [26]. Accordingly, absolute ID is diagnosed when serum ferritin levels are below 100 mg/L. Serum ferritin levels of 100–299 mg/L and TSAT of less than 20% confirm functional ID (Table 2).

**Table 2:** Diagnostic criteria for ID in HF based on the ESC guidelines

Diagnostic criteria for ID in HF based on the 2021 ESC HF guidelines	Potential new diagnostic criteria for ID in HF
Serum ferritin < 100 μg/l	TSAT < 20%
*or*	
Serum ferritin 100–299 μg/l	
*and*	
TSAT < 20%	

ID HF TSAT HFmrEF HFrEF ESC

Safety data on IV iron in patients with HF and hemoglobin > 15 g/dl is not available

The diagnostic criteria of ID in patients with HFmrEF and HFrEF based on the ESC guidelines 2021 and a possible new simplified criterion

*ESC* European Society of Cardiology, *HF* Heart Failure, *HFmrEF* Heart Failure with mildly reduced Ejection Fraction, *HFrEF* Heart Failure with reduced Ejection Fraction, *ID* Iron Deficiency, *IV* Intravenous, *TSAT* Transferrin Saturation

## Critical comments on the current diagnostic criteria

The HF patients often show functional ID, with ineffective oral iron therapy due to markedly reduced absorption and macrophage trapping. The use of IV iron supplementation is based on the assumption that ID (absolute or functional) affects ATP production in cardiomyocytes and impairs skeletal muscle function causing exercise intolerance and weakness [27].

While about 50% of HF patients meet the ferritin-based criteria for ID (serum ferritin < 100 µg/l or serum ferritin 100–299 µg/l and TSAT < 20%), only 20–25% have deficient myocardial iron stores [28], which possibly leads to an overestimation of true ID [29]. Established iron biomarkers do not reliably indicate myocardial ID and systemic iron does not correlate with cardiac iron content [27, 30]. In clinical practice, neither the measurement of hepcidin levels (poorly standardized assays) nor the identification of iron-overloaded macrophages in the bone marrow or hepatocytes (by magnetic resonance imaging, MRI), which reflects the iron status more accurately, are used routinely [31, 32]. Serum ferritin and hepcidin levels also seem unreliable for identifying ID in chronic heart failure (CHF) [33]. This is also reflected by various studies, indicating that the ferritin level in CHF does not predict outcome, whereas TSAT of less than 20% and serum iron levels below 300 µg/l does [34, 35].

Although a TSAT < 20% indicates deficient iron availability [22], it does not define an iron deficient state unless serum ferritin is < 300 μg/L according to current guidelines [26]. Conversely, patients with high ferritin (≥ 300 μg/L) but low iron (≤ 13 μmol/L), low TSAT (< 20%), or anemia who are at high risk are not identified by current guidelines. Of note, patients with ferritin < 100 μg/L but normal iron and TSAT have a better prognosis [36].

Meta-analyses show that IV iron supplementation benefits patients with TSAT < 20% but not with TSAT ≥ 20%. The IRONMAN [37] and AFFIRM-AHF [27] (with baseline TSAT ≈ 15%) studies demonstrated benefits from IV iron, whereas the HEART-FID trial (with baseline TSAT 23–24%) did not. Additionally, in HEART-FID, as well as in IRONMAN participants with isolated low ferritin levels did not show improved outcomes with IV supplementation [38]. This leads to the assumption that low TSAT but not low ferritin, indicates the effectiveness of IV iron to reduce HF hospitalizations.

In summary, the available evidence suggests that patients with isolated ferritin < 100 μg/L should not be considered for iron supplementation. This contradicts the recommendations of current guidelines. Ferritin levels < 20 μg/L indicate absolute deficiency but in CHF few patients even with anemia have isolated ferritin < 20 μg/L. Most patients with ferritin < 20 μg/L also have TSAT < 20%. Experience is limited with IV iron supplementation in patients with TSAT < 20% and ferritin > 400 μg/L, a rare profile in HF.

Efficacy analyses based on current eligibility criteria underestimate IV iron benefits. A meta-analysis of trials with FCM showed a reduction in cardiovascular (CV) death or HF hospitalizations by 13% in patient with guideline-recommended criteria and by 33% when only patients with TSAT < 20% and ferritin < 400 μg/L were considered [39]. For instance, the IRONMAN study showed a 20% risk reduction in patients with TSAT < 20%, versus 4% in those with ferritin < 100 μg/L and TSAT > 20%. This supports the view that ID should be better defined by TSAT < 20% and serum ferritin < 400 μg/L and serum ferritin < 100 μg/L should be discarded as the sole criterion.

## Treatment of ID in HF

Absolute ID usually responds to oral iron supplementation due to hepcidin suppression, while functional ID, with elevated hepcidin, requires intravenous (IV) iron administration. The use of IV iron rapidly increases cytosolic iron levels, even with intracellular trapping in ferritin [33].

Randomized controlled trials have demonstrated that IV iron supplementation in HF patients with reduced EF (HFrEF) improves symptoms, functional capacity, peak oxygen consumption, QOL and reduces the risk of first hospitalization for worsening HF.

Consequently, IV FCM and FDI is recommended to correct ID and improve clinical outcomes in HFrEF patients. Despite the recognition of the clinical significance of ID in HF, the effects of IV iron on morbidity and mortality are still under debate.

## Oral iron supplementation

Oral iron supplements are frequently used for the treatment of ID anemia due to their simplicity and cost-effectiveness.

Oral iron treatment generally yields favorable outcomes in patients with nutritional ID, bleeding anemia or in menstruating women without significant comorbidities; however, its efficacy is considerably lower in chronic diseases, such as renal anemia, rheumatic diseases and HF, where iron absorption and utilization may be impaired.

Oral iron supplementation has not shown benefits in patients with HF and reduced ejection fraction (IRONOUT trial), failing to improve any iron variable and thereby consequently also not exercise capacity, NT-proBNP levels and QOL [40].

Due to lack of efficacy in one controlled trial the current guidelines do not recommend the use of oral iron supplements in HF. Existing oral iron formulations require re-evaluation in light of recent advancements in dosing regimens and slow release technologies.

## IV iron supplementation

The origins of IV iron treatment in HF can be traced back to studies exploring the role of anemia management and the combined use of erythropoiesis stimulating agents (ESAS) and iron supplementation, which has been a standard combination in renal anemia. This has led to promising results in small trials; however, ESAS did not demonstrate significant clinical benefits in HF patients but increased rates of thromboembolic events and septic shock (Reduction of Events with Darbepoetin alfa in Heart Failure: RED-HF) [41], leading to a class III recommendation against its use.

**Fig. 1:**
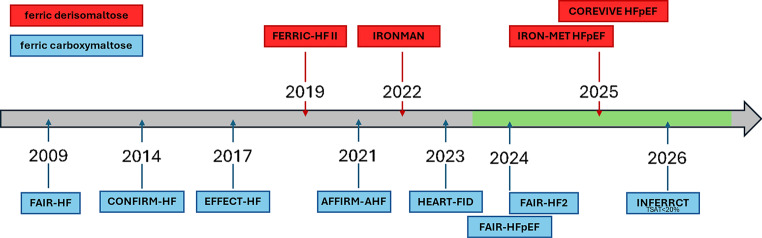
Chronological overview of major trials (with year of publication) and ongoing trials (with expected year of publication, *green timeline*) evaluating the effects of iron deficiency treatment in heart failure. Trials with ferric derisomaltose are marked in *red*, those with ferric carboxymaltose in *blue*. *FAIR-HF* Ferric Carboxymaltose in Patients with Heart Failure and Iron Deficiency; *CONFIRM-HF* Ferric Carboxymaltose evaluation on performance in patients with Iron deficiency in combination with chronic Heart Failure; *EFFECT-HF* Effect of Ferric Carboxymaltose on Exercise Capacity in Patients With Chronic Heart Failure and Iron Deficiency; *FERRIC-HF II* Effect of Iron Isomaltoside on Skeletal Muscle Energetics in Patients With Chronic Heart Failure and Iron Deficiency; *AFFIRM-AHF* Ferric carboxymaltose for iron deficiency at discharge after acute heart failure; IRONMAN:Intravenous ferric derisomaltose in patients with heart failure and iron deficiency in the UK; *HEART-FID* Ferric Carboxymaltose in Heart Failure with Iron Deficiency; *FAIR-HFpEF* Ferric carboxymaltose and exercise capacity in heart failure with preserved ejection fraction and iron deficiency; *FAIR-HF2* Intravenous iron in patients with systolic heart failure and iron deficiency to improve morbidity and mortality; *IRON-MET HFpEF* Impact of Intravenous Iron Repletion On Mechanisms of Exercise InTolerance in HFpEF; *COREVIVE HFpEF* The Effects of Ferric Derisomaltose in Patients with Acute Heart Failure and Iron Deficiency on Exercise Capacity and Quality of Life; *INFERRCT* Effect of Intravenous Ferric Carboxymaltose Onmortality and Cardiovascular Morbidity, and Quality of Life in Iron Deficient Patients With Recent Myocardial infarction

In contrast, iron supplementation alone without ESAS has emerged as a promising therapeutic option for HF patients with ID. Figure 1 and Table 3 provide an overview of randomized controlled trials (RCT) with FCM and FDI in patients with HF. The first prospective trial to demonstrate improved prognosis, QOL and exercise capacity with FCM in HF patients independent of anemia was FAIR-HF [42, 43].

The CONFIRM-HF trial [44, 45], which included 304 ambulatory HF patients (NYHA class II–III, LVEF ≤ 45%, elevated natriuretic peptides), confirmed the long-term safety and efficacy of IV iron. Patients with absolute or functional ID (serum ferritin < 100 µg/L or 100–299 µg/L with TSAT < 20%, Table 2) were randomized to receive IV FCM or placebo for 52 weeks. The IV iron dosage was individualized based on body weight and hemoglobin levels (Table 4). The primary endpoint, a change in 6‑minute walk distance (6MWD) at 24 weeks, showed a significant improvement of 33 m in the IV iron group compared to placebo. This benefit persisted through 52 weeks and was consistent across all predefined subgroups. Secondary endpoints, including NYHA class, patient global assessment (PGA), QOL, and HF-related hospitalizations, also significantly improved with IV iron treatment. The average dose required was 1500 mg. The study reported no difference in mortality or adverse events between the two groups.

**Table 3:** Overview of selected randomized trials investigating IV iron treatment in HF

Trial	Eligibility criteria	Design/dosage	Primary endpoint	Secondary endpoints
FAIR-HF Ferric carboxymaltose [43]	NYHA II + LVEF ≤ 40% or ≤ 45% + NYHA III; ferritin < 100 ng/ml or 100–299 ng/ml + TSAT < 20%, Hb 9.5–13.5 g/dl	2:1 randomization, IV FCM or placebo. 200 mg IV weekly (correction phase), 200 mg IV 4‑weekly (maintenance phase). *N* = 459	Patient global Assessment (PGA) (*p* < 0.001) and NYHA (*p* < 0.001) at week 24	6 MWD (*p* < 0.001), QOL (*p* < 0.001)
CONFIRM-HF [45]	NYHA II–III, LVEF ≤ 45%, ferritin < 100 µg/L or 100–299 µg/L with TSAT < 20%	1:1 randomization to IV FCM or placebo for 52 weeks. *N* = 304	Change in 6MWD at 24 weeks (+33m) (*p* = 0.002)	↑NYHA class, PGA, QOL, and ↓HF hospitalizations significantly
EFFECT-HF [47]	NYHA II–III, LVEF ≤ 45%, with ID	IV FCM for 24 weeks or SOC. *N* = 172	Improvement in maximum oxygen uptake (VO_2_) at 24 weeks	IV FCM significantly ↑serum ferritin and TSAT after 24 weeks, ↑PGA and ↑NYHA
AFFIRM-AHF [27] Ferric carboxymaltose	Acute HF-hospitalization + LVEF < 50%; ferritin < 100 ng/ml or 100–299 ng/ml + TSAT < 20%	1 dose at discharge, FCM at week 6, 12, 24. *N* = 1108	CV death or HF hospitalization over 52 weeks (*p* = 0.059; *p* = 0.024 in COVID analysis)	Time to first HF hospitalization or CV death (*p* = 0.050); reduction in HF hospitalizations (*p* = 0.013)
IRONMAN [37] Ferric derisomaltose	NYHA II–IV, LVEF ≤ 45%, recent HF hospitalization or NT-proBNP > 250/1000 ng/L; ferritin < 100 ng/mL or TSAT < 20%, Hb 9–13 (female), 9–14 (male)	Dose adaptation to BW and Hb: 20 mg/kg–2000 mg. *N* = 1137	HF hospitalizations and CV death (*p* = 0.07); significant benefit in COVID-19 analysis (*p* = 0.047)	↑QOL, greatest benefit in patients with TSAT < 20%. Long-term safety
HEART-FID [48] Ferric carboxymaltose	HF hospitalization (12m) or NT-proBNP > 600/ > 1000 ng/l if AF, LVEF ≤ 40% ferritin < 100 ng/ml or 100–299 ng/ml + TSAT < 20%, Hb 9–13 g/dL (females) or < 15 g/dL (males)	Dose every 6 m based on labs: < 50 kg–2 doses of 15 mg/kg, ≥ 50 kg–2 doses of 750 mg (separated by 7 days). *N* = 3065	Hierarchical composite: All-cause mortality, HF hospitalizations (12 months), 6MWD (6m); (*p* = 0.019, ns)	CV death or first HF hospitalization (ns)
FAIR-HFpEF [49] Ferric carboxymaltose	LVEF ≥ 45%, NYHA II-III + treatment with diuretic, HF hospitalization (12m) or NT-proBNP > 300/ > 600 ng/l if AF, ferritin < 100 ng/ml or < 300 ng/ml + TSAT < 20%, 6MWD < 450m	Dose 0, 1, 16, 32 weeks. *N* = 39	Trial stopped due to slow recruitment. Change in 6MWD after 24 weeks (*p* = 0.029)	Secondary endpoints ns

This table provides an overview and does not claim to be exhaustive

*FCM* ferric carboxymaltose, *NYHA* New York Heart Association functional classification, *LVEF* left ventricular ejection fraction, *TSAT* transferrin saturation, *Hb* hemoglobin, *SOC* standard of care, *6MWD* Six-minute walk distance, *PGA* patient global assessment, *QOL* quality of life, *CV* cardiovascular, *HF* heart failure, *ID* iron deficiency, *NT-proBNP* N-terminal pro-B-type natriuretic peptide, *AF* atrial fibrillation, *BW* body weight, *ns* not significant, *IV* intravenous

**Table 4:** Calculation of total iron requirement for FCM substitution according to authorization in Austria (Z. Nr.: 1‑27299) [29] (**a**) and dosing of FDI according to IRONMAN [37] (**b**)

Hb		Patient body weight		
**(a) Determination of total iron requirement (FCM) **				
*g/dL*	*mmol/L*	*Below 35* *kg*	*35 to <* *70* *kg*	*70* *kg and above*
< 10	< 6.2	500 mg	1500 mg	2000 mg
10–14	6.2–8.7	500 mg	1000 mg	1500 mg
> 14	> 8.7	500 mg	500 mg	500 mg
**(b) Dose regimen for FDI according to IRONMAN** [37]				
*g/dL*	*mmol/L*	*Below 50* *kg*	*50 to <* *70* *kg*	*70* *kg and above*
< 10	≥ 6.2–8.7	20 mg/kg	20 mg/kg	20 mg/kg–max. 2g
≥ 10	< 6.2	20 mg/kg	1000 mg	20 mg/kg–max. 1.5 g

A single FCM administration should not exceed:

– 15 mg iron/kg body weight (for administration by IV injection) or 20 mg iron/kg body weight (for administration by IV infusion)

– 1000 mg of iron

The maximum recommended cumulative dose of FCM is 1000 mg of iron (20 mL) per week.

A single infusion of FDI or the total dose per week should not exceed a dose of 20 mg iron/kg body weight

The subsequent EFFECT-HF study (*n* = 172) demonstrated a positive effect of IV FCM treatment on the exercise capacity of symptomatic patients with HF and ID [47]. After 24 weeks there was a statistically significant difference in maximum oxygen uptake (VO_2_) for IV iron substitution compared to standard treatment, irrespective of the presence of anemia [47].

The recently published studies IRONMAN [37], HEART-FID [48] and AFFIRM-AHF [50] provide important additional information on the benefits of IV iron early after acute worsening of HF and demonstrating a beneficial effect not only for FCM but also for FDI in HF. Figure 1 provides an overview of RCT evaluating FCM und FDI in HF.

The AFFIRM-AHF (*n* = 1132) study was the first RCT to evaluate the effects of IV FCM administration initiated at discharge, on mortality and morbidity in HF patients with ID hospitalized for an acute HF episode. The primary analysis tended to show a beneficial effect of IV FCM on its combined endpoint of HF hospitalization and CV death (RR 0.79; 95% confidence interval, CI 0.62–1.01; *p* = 0.059). Due to coronavirus disease 2019 (COVID-19), the European Society of Cardiology – Heart Failure Association, the European Medicines Agency, and the US Food and Drug Administration (FDA) recommended a pre-COVID-19 sensitivity analysis. This analysis censored patients at the date when the first COVID-19 patient occurred and showed a significant 25% reduction in the relative risk (RR) for the primary endpoint (RR 0.75; 95% CI 0.59–0.96; *p* = 0.024). This outcome was driven by a significant 26% reduction in total HF hospitalizations (*p* = 0.013), whereas there was no difference in CV deaths. Significant benefits were also observed in time to first HF hospitalization or CV death. Notably, 80% of patients required only 1–2 FCM injections and the treatment was well tolerated [27].

The IRONMAN trial (*n* = 1137) addressed the long-term effects of IV FDI, with a median follow-up period of 2.7 years. This investigator-initiated, prospective, open-label trial enrolled patients with a left ventricular ejection fraction (LVEF) ≤ 45% and either TSAT < 20% or serum ferritin < 100 μg/L. The IRONMAN trial, which employed a blinded-endpoint design, primarily enrolled ambulatory patients, with 14% of participants being recruited during a hospitalization for HF and 18% having experienced an HF hospitalization in the preceding 6 months. Patients with hemoglobin levels > 13 g/dL (for women) and > 14 g/dL (for men) were excluded from the study. The primary endpoint of the trial was a composite of total (first and recurrent) HF hospitalizations and CV death. The RR for this endpoint was 0.82 (95% CI 0.66–1.02; *p* = 0.070) and similar to the sensitivity analysis of AFFIRM-AHF a prespecified sensitivity analysis accounting for the COVID-19 pandemic demonstrated a statistically significant benefit (RR 0.76, 95% CI 0.58–1.00; *p* = 0.047). Additionally, IV iron appeared to improve QOL, with patients with TSAT < 20% deriving the greatest benefit. Moreover, the IRONMAN trial provided evidence that IV iron supplementation with FDI has a reassuring long-term safety profile in HF patients [37, 51].

The HEART-FID trial enrolled 3065 patients with HF who had either been hospitalized for HF within the previous 12 months or had elevated NT-proBNP levels (> 600 ng/L in those with sinus rhythm or > 1000 ng/L in those with atrial fibrillation). The ID was defined as a serum ferritin level < 100 ng/mL or between 100 and 299 ng/mL plus a TSAT < 20%. The FCM was administered every 6 months, with dosing based on body weight and laboratory results. Patients weighing less than 50 kg received 2 doses of 15 mg/kg body weight, while those weighing 50 kg or more received two doses of 750 mg, with the doses separated by a 7-day interval. A total of 3014 patients were included in the trial. The primary endpoint was a hierarchical composite of all-cause mortality, HF hospitalizations over 12 months and the 6MWD at 6 months. This primary endpoint was marginally but not statistically improved (*p* = 0.019) missing the *p*-value set at < 0.01. Secondary endpoints included CV death and HF hospitalizations which differed nonsignificantly [48].

In a meta-analysis by Graham et al. including 10 trials with 3373 patients with CHF and acute heart failure (AHF) and LVEF ≤ 45%, IV iron reduced the composite of total HF hospitalizations and CV death (RR 0.75, 95% CI 0.61–0.93; *P* < 0.01) and time to first HF hospitalization or CV death (odds ratio, OR 0.72, 95% CI 0.53–0.99; *p* = 0.04). There was no effect on CV (OR 0.86, 95% CI 0.70–1.05; *p* = 0.14) or all-cause mortality (OR 0.93, 95% CI 0.78–1.12; *p* = 0.47).

The IRON-CRT study examined the impact of IV FCM on heart remodelling and contractility in patients with ID and LVEF < 45%. Out of 75 patients, those on FCM showed a significant LVEF improvement (+4.22%) versus standard of care (−0.23%, primary endpoint) after 3 months. A reduction in left ventricular end-systolic volume (LVESV) but not in end-diastolic volume (LVEDV) was observed. The use of FCM also improved cardiac performance which was indicated by a better force-frequency relationship, moving from a negative to a positive slope in the cardiac contractility index (systolic blood pressure/LVESV index). Functional status and exercise capacity also improved with FCM [52].

In the most recent FAIR-HFpEF trial involving patients with ID and HF with preserved ejection fraction (HFpEF), IV FCM improved 6MWD and was associated with fewer serious adverse events [49]; however, the trial was stopped because of slow recruitment after 39 patients and was therefore underpowered to assess effects on symptoms or QOL. Further research in a larger cohort is needed to confirm the benefits of IV iron in this population.

## Adverse effects and complications of IV iron treatment

For decades, IV iron was regarded as dangerous, primarily due to the risk of anaphylaxis, a rare but serious reaction associated with high molecular weight iron dextran formulations, first approved in 1977, which are no longer in use [53]. In recent years, the number of available IV iron agents has expanded, with the introduction of sodium ferric gluconate (1999) and iron sucrose (2000), followed by ferumoxytol (2009) and more recently FCM and FDI. Newer formulations utilize carbohydrate cores that tightly bind elemental iron, reducing its release into the circulation. This advancement enables complete iron replacement within a single 15–60-min session. Characteristics of the third generation IV iron formulations are detailed in Table 5, [54].

**Table 5:** Third generation IV iron formulations

Agent	Carbohydrate	Molecular weight, Da	Concentration of elemental iron (mg/mL)	Maximum single dose (mg)	Maximum dose/week (mg)
Ferric carboxymaltose (FCM)	Carboxymaltose	150,000	50	1000	1000
Ferric derisomaltose (FDI)	Isomaltoside	150,000	100	2000	2000

Newer iron formulation recommended by current ESC guidelines with carbohydrate cores. These formulations bind elemental iron more tightly and potentially allow complete iron replacement within 15 min in one visit

Hypersensitivity reactions to IV iron are rare and most commonly occur within the first 10 min of infusion. These reactions are categorized into two main types: the self-limiting Fishbane type reactions, which vary in presentation and severity without anaphylactic symptoms, characterized by transient flushing, truncal myalgia and/or chest tightness that spontaneously resolve within minutes. This reaction type is thought to be triggered by labile or free iron and has been observed with all IV iron formulations and it typically resolves without intervention once the IV iron infusion is stopped and does not usually recur upon rechallenge. In contrast, anaphylactic reactions, marked by the acute onset of skin and/or mucosal symptoms, gastrointestinal distress, respiratory involvement and/or hypotension, require immediate and appropriate intervention [55].

There is an ongoing debate regarding the potential for exacerbating bacterial infections, as iron is a critical nutrient for bacterial growth [56]; however, neither the AFFIRM study with FCM [50] nor the IRONMAN study [37] with FDI showed any signs of an increase in infection rates.

In two meta-analyses of randomized clinical trials various IV iron formulations were compared with oral and intramuscular iron, placebo or no iron. Both analyses demonstrated that IV iron has a comparable safety profile to placebo, with no increased risk of serious adverse effects (SAE) or infections. Gastrointestinal events were less frequent with IV iron compared to oral iron. Although serious infusion reactions were extremely rare, they did occur (35 out of 9223 patients) [57, 58]. Remarkably, a subgroup analysis indicated a reduction in SAEs when IV iron was used in HF compared to oral supplementation [57].

In a meta-analysis by Avani et al. serious infusion reactions were particularly increased with ferric gluconate compared to placebo, oral, intramuscular and no iron (RR 5.32; 95% CI 1.49–18.99) [57]. In this analysis other iron preparations were not associated with a statistically significant increased risk of severe infusion reactions (iron dextran: RR 3.1; 95% CI 0.86–11.22; ferumoxytol: RR 2.26; 95% CI 0.19–26.22; iron sucrose: RR 1.75; 95% CI 0.69–4.43; FCM: RR 1.47; 95% CI 0.40–5.39; FDI: RR 1.00; 95% CI 0.99–1.01).

The two IV iron formulations, FCM and FDI, which were tested in HF and approved for the administration of a sufficient single dose to correct a substantial ID were directly compared for infusion reactions in four analyses: two single-center cohort studies including 1332 and 231 patients showed a lower risk for hypersensitivity of FCM compared to FDI [59, 60]. In contrast, an analysis which included 21 prospective trials with more than 8000 patients found a decreased risk of serious or severe hypersensitivity reactions with FDI compared to FCM [61]; however, the overall rate of hypersensitivity reactions is low and severe hypersensitivity is very rare for either formulation used in HF [62].

Hypophosphatemia (HPP) is a known adverse effect of iron infusions, particularly with FCM. It is essential for clinicians to recognize the signs of HPP associated with iron infusion, which is characterized by hyperphosphaturic hypophosphatemia, low 1,25-dihydroxyvitamin D2, hypocalcemia and secondary hyperparathyroidism.

The clinical significance of FCM-induced HPP in HFrEF is currently under debate. A substudy of the HEART-FID study examined serum phosphate, 1,25-dihydroxyvitamin D2 and parathorme (PTH) after FCM infusion. This substudy measured changes in serum phosphate, vitamin D metabolites, and PTH over 6 months. Among 133 patients (62 FCM, 71 placebo), phosphate levels dropped in 57.6% of FCM patients versus 10.3% in the placebo group, with a nadir at day 21 and moderate to severe HPP in 51%. Reductions in 1,25-dihydroxyvitamin D and increases in PTH were observed but levels returned to baseline by day 91. No serious adverse events were reported in this short follow-up [63].

In contrast, Frezier et al. reported on a cohort of 16 women treated for ID anemia at a single-center hematology clinic, receiving a twice weekly dose of FCM. The study observed significant renal phosphate wasting, leading to hypophosphatemia in 87.5% of the patients, which persisted for over 5 weeks. Similarly, Stöhr et al. reported that 60.8% (14 out of 24) of patients with HFrEF developed significant (transient) HPP following high-dose FCM administration. This finding suggests worse laboratory derangements with multiple infusions or high dosages and also raises concerns about potential long-term effects on bone mineralization specifically in older woman with an even higher risk of osteoporosis [64, 65].

Comparative clinical studies indicated that serum phosphate levels decrease more frequently following FCM infusion compared to FDI [60]. Schafer et al. analyzed data from the PHOSPHARE-IDA trials, which included 245 patients with ID anemia who were treated with either FCM or FDI. The study concluded that FCM was associated with a significantly higher risk of hypophosphatemia compared to FDI (OR: 38.37, *P* < 0.001), with 40% of patients in the FCM group demonstrating persistent hypophosphatemia by day 35 [66].

A pooled analysis of 45 clinical trials with FCM showed that while FCM induces a temporary decrease in serum phosphate levels, this effect generally resolves within 4–12 weeks [67]. The proportion of patients with moderate (1–> 2 mg/dL) and severe (< 1 mg/dL) HPP was highest in gastroenterology (47.1%) and neurology (39%) patients and lower in cardiology (9.9%), non-dialysis-dependent chronic kidney disease (12.3%) and hemodialysis patients (0%). Of 49 patients with severe HPP, 55.1% were in gastroenterology studies and 22.4% in women’s health studies, highlighting that the primary risk factors for severe HPP are treatment area (gastroenterology) and a cumulative (single or repeated) FCM dose of > 1000 mg within the first 4 weeks of treatment. Notably, the maximum single dose did not correlate with a severe HPP risk and no patient with a serum PO_4_^3−^ level < 1 mg/dL had an adjudicated serious adverse event.

Regarding HF, it has to be mentioned that repeated doses are rare and HF is a condition with a trend to higher levels of serum phosphate in advanced stages [68].

Symptomatic HPP may present as fatigue, myalgia, or bone pain, with cases of hypophosphatemic osteomalacia reported in patients with pre-existing risk factors who received high cumulative doses or prolonged FCM treatment [69–71]. Monitoring serum PO_4_^3−^ levels in patients receiving repeated doses of IV FCM is therefore prudent [67].

The efficacy of vitamin D supplementation prior to administering FCM remains uncertain. We agree that FCM-induced severe HPP should be treated with IV phosphate supplementation, although the evidence remains sparse [72]. Ultimately, in patients with confirmed occurrence of FCM-induced HPP, switching to FDI for future administrations appears advisable [73].

## Dosage of IV iron treatment and follow-up evaluation

Serum iron levels and TSAT quickly increase after IV iron supplementation but the clinical significance of these short-term changes is unclear. Oral iron also raises serum iron and TSAT without clinical benefits, likely due to slow intracellular iron increases. In anemic patients the first physiological response to iron supplementation in erythroblasts is a rise in reticulocyte hemoglobin, indicating enhanced hemoglobin synthesis in new red blood cells [74]. This rise occurs within 24–48 h following IV iron administration, whereas hemoglobin increase takes weeks and may be modest in nonanemic patients [75]; however, it is uncertain if an erythroid response indicates cytosolic iron repletion in cardiomyocytes. An instant increase in the phosphocreatine/ATP ratio by magnetic resonance spectroscopy after IV iron suggests alleviation of cardiomyocyte ID but such measurements are not yet performed [39].

The most widely used parameter for determining iron status after IV iron treatment is an increase in serum ferritin. Ferritin levels rise significantly as the body produces ferritin to prevent oxidative damage from excess iron [76]. In the IRONMAN study the ferritin levels increased to over 500 µg/L after 4 weeks, while the AFFIRM-AHF study showed an increase to about 350 µg/L. Excessive ferritin levels above 400 µg/L may indicate short-term iron overload, which is associated with iron accumulation in the liver and cell damage. Vera-Aviles et al. recently demonstrated that non-transferrin-bound iron, after IV iron supplementation, is rapidly delivered to the myocardium without the involvement of reticuloendothelial macrophages. Due to the heart’s limited iron storage capacity, this iron remains labile for weeks, raising concerns about the potential for cumulative iron accumulation with long-term dosing [77].

It should also be noted that sodium-glucose transporter 2 (SGLT2) inhibitors and neprilysin inhibitors reduce ferritin but they can mobilize iron from stores to cover the demand. Thus, ferritin levels may not reliably indicate iron status in these cases [78, 79].

Because of these diagnostic uncertainties the dosing of iron supplementation in HF should be based on clinical outcome data derived from RCT rather than solely on biochemical measurements. Functional ID may persist even after iron supplementation, raising the possibility that HF patients could need ongoing iron treatment, even if iron levels appear adequate. As the dosing regimens in the various studies, especially for FCM, varied widely, it seems difficult to establish clear standards. For FDI, the recommendation is based on the IRONMAN dosing scheme. Table 4 provides an overview of the recommended dosages.

**Fig. 2:**
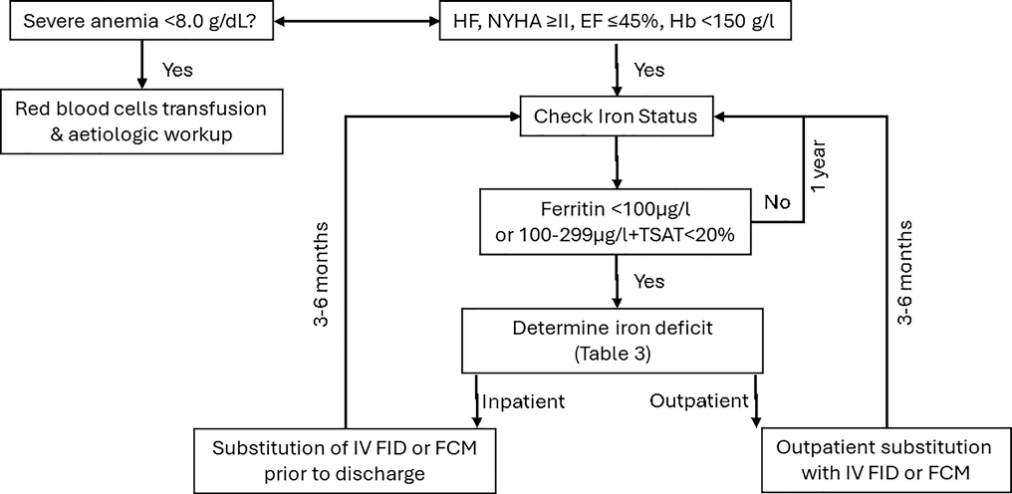
Recommended management of iron deficiency in heart failure. *EF* Ejection Fraction, *FCM* Ferric Carboxymaltose, *FID* Ferric Isomaltose, *Hb* Hemoglobin, *HF* Heart Failure, *IV* Intravenous, *NYHA* New York Heart Association, *TSAT* Transferrin Saturation

Regardless of the presence of HF an etiological assessment and red blood cell transfusion are indicated in cases of severe anemia. In patients with symptomatic HFrEF and hemoglobin (Hb) < 150 g/L, it is recommended that the iron status should be assessed according to current guidelines (Fig. 2; [26]).

If ID is confirmed, inpatients should receive iron replacement during the hospital stay based on the results of the AFFIRM-AHF trial. For outpatients, iron supplementation should be planned in a timely manner after confirmation.

After IV iron administration the iron status should be reassessed in 3–6 months. If the results are normal, routine annual checks are recommended.

## Current recommendations

The 2021 European Society of Cardiology (ESC) guidelines on HF highlight the significance of ID and provide recommendations for its diagnosis and treatment (Table 6). The focused update of 2023 [80] recommends IV iron supplementation in symptomatic patients with HFrEF and HFmrEF and ID to alleviate HF symptoms and improve QOL.

**Table 6:** Recommendations for the treatment of other comorbidities in patients with HF considering the ESC guidelines 2021 and the update 2023 [26, 80]

Recommendations ID	Class	Level of evidence
IV iron supplementation is recommended in symptomatic patients with HFrEF and HFmrEF and ID to alleviate HF symptoms and improve QOL [80]	I	A
It is recommended that all patients with HF are periodically screened for anemia and ID with a full blood count, serum ferritin concentration, and TSAT [26]	I	C
IV iron supplementation with FCM or FDI should be considered in symptomatic patients with HFrEF and HFmrEF, and ID, to reduce the risk of HF hospitalization [80]	IIa	A
“Treatment of anemia in HF with erythropoietin stimulating agents is not recommended in the absence of other indications for this therapy” [26]	III	–

ID HFrEF etc.

Oral iron therapy is not effective in replenishing iron stores and has not improved performance in patients with HFrEF and ID. It is therefore not recommended for the treatment of ID in patients with HF

*FCM* Ferric Carboxymaltose, *FDI* Ferric Derisomaltose, *HF* Heart Failure, *HFmrEF* Heart Failure with mildly reduced Ejection Fraction, *HFpEF* Heart Failure with preserved Ejection Fraction, *HFrEF* Heart Failure with reduced Ejection Fraction, *ID* iron deficiency, *IV* intravenous, *QOL* Quality Of Life, *TSAT* Transferrin Saturation

Given the discussion above, simplifying the diagnostic criteria to a TSAT < 20%, may be considered (Table 2); however, this represents a well-argued post hoc expert opinion not covered by the ESC guidelines. The ID remains underrecognized and undertreated in clinical practice, partly due to a lack of practical guidance for clinicians. Figure 2 shows a potential management regimen for the screening and treatment of iron insufficiency in HF.

The supplementation FCM or FDI should be considered in symptomatic patients with HFrEF, HFmrEF and ID to reduce the risk of hospitalization. Considering its lower association with hypophosphatemia, FDI may be preferred in patients at risk for hypophosphatemia and osteoporosis, when repeated infusions are required, although adverse outcomes in HF patients due to hypophosphatemia using FCM remain ill-defined.

Given the limited data, current guidelines do not provide a specific recommendation for IV iron supplementation in HFpEF.

## Outlook for the future

The rapid increase in evidence has led to an adaptation of the guidelines. Since 2021 there are new recommendations for screening and diagnosis of ID [80]. Treatment is recommended to improve functional status and QOL. Data on the efficacy of IV iron supplementation in HFpEF patients are expected in the next years.

Other iron formulations and delivery methods are currently under review; however, none have yet achieved sufficient evidence for recognition or endorsement. There is a critical need to refine current approaches by developing more precise diagnostic criteria for tissue-specific ID and by targeting iron homeostasis pathways more effectively [81, 82].

## Take home messages

- ID in HF is common and associated with worse symptoms, reduced exercise capacity, higher hospitalization rates and increased mortality.
- Supplementation with FCM or FDI is recommended in symptomatic patients with HFrEF and HFmrEF and ID to alleviate HF symptoms and improve QOL and should be considered to reduce the risk of HF hospitalization. Data are needed to support IV iron efficacy in HFpEF.
- Current guidelines indicate iron supplementation in HF with EF < 45% and ID defined as serum ferritin < 100 µg/L or 100–299 µg/L with TSAT < 20%. The potential simplification of diagnostic criteria to TSAT < 20% is currently being discussed.
- Current guidelines recommend that all HFrEF or HFmrEF patients, regardless of glomerular filtration rate or hemoglobin level, should be screened for ID. It is recommended to screen for ID in HF patients at least annually, with particular attention to those classified as NYHA classes II–IV.
- IV iron formulations are effective in HF patients leading to a rapid correction of iron stores, while oral iron is poorly absorbed and has shown no benefit. FCM and FDI are approved for the administration of a single dose sufficient to correct ID.
- Follow-up: reassess iron parameters (serum ferritin and TSAT) 3–6 months post-infusion. Repeat dosing may be necessary if ID persists or reoccurs. Reassessment of iron status is recommended every 6–12 months in patients initially presenting without ID.
- Infusion reactions with FCM and FDI are rare. Hypophosphatemia occurs more frequently with FCM and should be monitored.
- Integration of testing for ID and IV iron supplementation into HF management programs is recommended.
