# Correction to: Diagnosis and treatment of iron deficiency in chronic heart failure

**DOI:** 10.1007/s00508-025-02576-w

**Published:** 2025-08-01

**Authors:** Moritz Messner, Gerhard Pölzl, Christopher Adlbrecht, Johann Altenberger, Johann Auer, Robert Berent, Jakob Dörler, Marc-Michael Zaruba, Christian Ebner, Friedrich Fruhwald, Martin Hülsmann, Deddo Mörtl, Peter P. Rainer, Anna Rab, Thomas Weber, Rudolf Berger, The Heart Failure Working Group of The Austrian Society for Cardiology

**Affiliations:** 1 Department of Internal Medicine III, Medical University Innsbruck, Innsbruck, Tyrol Austria; 2 Imed19-privat, private clinical research center, Chimanistrasse 1, 1190 Vienna, Austria; 3 Center for Cardiovascular Rehabilitation, Lehrkrankenhaus der PMU, Pensionsversicherung Grossgmain, Grossgmain, Austria; 4 Department of Cardiology and Intensive Care, St Josef Hospital, Braunau, Upper Austria Austria; 5 Center for Cardiovascular Rehabilitation, HerzReha Bad Ischl, Bad Ischl, Upper Austria Austria; 6 Department of Internal Medicine and Cardiology, Landeskrankenhaus Klagenfurt, Klagenfurt, Carinthia Austria; 7 Second Medical Department, Convent Hospital Elisabethinen, Linz, Upper Austria Austria; 8 Department of Internal Medicine, Division of Cardiology, Medical University Graz, Graz, Styria Austria; 9 University Clinic of Internal Medicine II, Department of Cardiology, Medical University Vienna, Vienna, Austria; 10 Department of Internal Medicine 3, University Hospital St. Poelten, Karl Landsteiner Private University, St. Poelten, Lower Austria Austria; 11 Department of Internal Medicine, St. Johann in Tirol General Hospital, St. Johann in Tirol, Austria; 12 University Heart Center, Medical University of Graz, Graz, Austria; 13 BioTechMed Graz, Graz, Austria; 14 Department Internal Medicine I, Kardinal Schwarzenberg Klinikum, Schwarzach, Austria; 15 Department of Cardiology, Klinikum Wels-Grieskirchen, Wels-Grieskirchen, Upper Austria Austria; 16 First Medical Department, Hospital of St. John of God, Eisenstadt, Burgenland Austria

**Correction to:**

**Wien Klin Wochenschr 2025**

10.1007/s00508-025-02521-x

In the original version of this article, Table 4 was incorrectly given as:

Table 4 (original)

**Table 4 (original):** Calculation of iron supplementation dosage for (**a**) single administration of FCM according to authorization in Austria (Z. Nr.: 1‑27299) [29] and for FDI in (**b**)

Hb		Patient body weight		
**(a) FCM**				
*g/dL*	*mmol/L*	*Below 35* *kg*	*35 to <* *70* *kg*	*70* *kg and above*
< 10	< 6.2	500 mg	1500 mg	2000 mg
10–14	6.2–8.7	500 mg	1000 mg	1500 mg
> 14	> 8.7	500 mg	500 mg	500 mg
**(b) Dose regimen for FDI according to IRONMAN** [46]				
*g/dL*	*mmol/L*	*Below 50* *kg*	*50 to <* *70* *kg*	*70* *kg and above*
< 10	< 6.2	20 mg/kg	1000 mg	20 mg/kg–max. 1.5 g
≥ 10	≥ 6.2–8.7	20 mg/kg	20 mg/kg	20 mg/kg–max. 2g

A single FCM administration should not exceed:

– 15 mg iron/kg body weight (for administration by IV injection) or 20 mg iron/kg body weight (for administration by IV infusion)

– 1000 mg of iron

The maximum recommended cumulative dose of FCM is 1000 mg of iron (20 mL) per week.

A single infusion of FDI or the total dose per week should not exceed a dose of 20 mg iron/kg body weight

and should have been:

Table 4 (corrected)

**Table 4 (corrected):** Calculation of total iron requirement for FCM substitution according to authorization in Austria (Z. Nr.: 1‑27299) [29] (**a**) and dosing of FDI according to IRONMAN [37] (**b**)

Hb		Patient body weight		
**(a) Determination of total iron requirement (FCM) **				
*g/dL*	*mmol/L*	*Below 35* *kg*	*35 to <* *70* *kg*	*70* *kg and above*
< 10	< 6.2	500 mg	1500 mg	2000 mg
10–14	6.2–8.7	500 mg	1000 mg	1500 mg
> 14	> 8.7	500 mg	500 mg	500 mg
**(b) Dose regimen for FDI according to IRONMAN** [37]				
*g/dL*	*mmol/L*	*Below 50* *kg*	*50 to <* *70* *kg*	*70* *kg and above*
< 10	≥ 6.2–8.7	20 mg/kg	20 mg/kg	20 mg/kg–max. 2g
≥ 10	< 6.2	20 mg/kg	1000 mg	20 mg/kg–max. 1.5 g

A single FCM administration should not exceed:

– 15 mg iron/kg body weight (for administration by IV injection) or 20 mg iron/kg body weight (for administration by IV infusion)

– 1000 mg of iron

The maximum recommended cumulative dose of FCM is 1000 mg of iron (20 mL) per week.

A single infusion of FDI or the total dose per week should not exceed a dose of 20 mg iron/kg body weight

The original article has been corrected.

